# Binge-eating disorder treatment goes online – feasibility, usability, and treatment outcome of an Internet-based treatment for binge-eating disorder: study protocol for a three-arm randomized controlled trial including an immediate treatment, a waitlist, and a placebo control group

**DOI:** 10.1186/s13063-019-3192-z

**Published:** 2019-02-13

**Authors:** Simone Munsch, Andrea Wyssen, Pierre Vanhulst, Denis Lalanne, Sharon T. Steinemann, Alexandre Tuch

**Affiliations:** 1 Department of Clinical Psychology and Psychotherapy, University of Fribourg, Rue de Faucigny 2, 1700 Fribourg, Switzerland; 2 Human-IST Institute, University of Fribourg, Boulevard de Perolles 90, 1700 Fribourg, Switzerland; 3 Department of General Psychology and Methodology, University of Basel, Missionsstrasse 62a, 4055 Basel, Switzerland; 4 Swiss Health Observatory, Federal Statistical Office, Espace de l’Europe 10, 2010 Neuchâtel, Switzerland

**Keywords:** Binge-eating disorder, Internet-based treatment, Cognitive behavioral therapy, Guided self-help, Placebo

## Abstract

**Background:**

Binge-eating disorder (BED) is characterized by recurrent episodes of loss of control over eating and is related to a higher prevalence of other mental disorders and somatic consequences associated with overweight and obesity. In community-based samples, 2–4% of women and 1–3% men are diagnosed with BED. Psychotherapeutic interventions focusing on maintenance factors of disturbed eating behavior have proven to be effective. However, treatment access is limited for a considerable number of patients with BED. A lack of specialized institutions and treatment resources, but also long distances to treatment facilities for people living in remote or rural areas are often causes of insufficient care. Internet-based guided self-help (GSH) programs have the potential to fill this gap.

**Methods:**

This project aims to develop and evaluate an Internet-based treatment for BED derived from an evidence-based manualized cognitive behavioral therapy (CBT). The primary goal is to test feasibility and suitability of the Internet-based program and to evaluate the treatment outcome in comparison to a pure and a placebo-inspired waitlist control group (i.e. reduction of binge-eating episodes and eating disorder pathology as primary outcome variables). In total, 60 women and men aged 18–70 years with a BED diagnosis will be recruited. The Internet-based GSH treatment comprises eight sessions followed by three booster sessions. The placebo-inspired waitlist control group receives weekly messages containing information increasing positive expectations regarding the treatment effects during the four-week waiting period. The pure waitlist control group receives weekly messages simply asking patients to fill in a short questionnaire.

**Discussion:**

The access to evidence-based treatments for BED might be made easier using an Internet-based GSH approach. The present study protocol presents a randomized controlled trial. As well as evaluating the suitability and efficacy of the Internet-based GSH treatment, there will also be a prelimarily investigation on the influence of positive expectations (placebo) for a therapeutic intervention on core symptoms.

**Trial registration:**

German Clinical Trials Register, DRKS00012355. Registered on 14 September 2017.

## Background

Binge-eating disorder (BED) is characterized by the experience of loss of control over eating, often accompanied by continuous weight gain, and is related to increased risk of the development of other mental disorders and somatic consequences associated with overweight and obesity [1]. While there are data about the course and outcome of eating disorders such as anorexia nervosa (AN) or bulimia nervosa (BN) in the general population, much less data are available on BED [2]. Besides elevated co-morbidity, current research highlights an increased risk of suicide even when accounting for co-morbidity [3]. In a community-based sample of 10,038 women and men in Switzerland aged 15–60 years, lifetime prevalence of any eating disorder (ED) was 3.5%. A total of 2.4% of women and 0.7% of men were diagnosed with BED, from which only 53.4% reported to have consulted a specialist due to their eating or weight regulation problems [4]. These measures are comparable with prevalence rates in other countries in Europe and somewhat lower than in the US [2, 3, 5]. in the absence of an appropriate treatment, the course of BED is often persistent (average illness duration of 5.79 years; SD = 8.45) in BED patients in Switzerland [4] and 4.3 years (range = 1–11.7 years) in a multinational study [6] and 8.1 years (SD = 1.1) in a US sample [7].

Even though BED represents a severe mental health condition, which can be treated efficaciously, treatment-seeking behavior and the access to treatment are limited. A recent study names the following essential barriers to treatment seeking of individuals suffering from BED: feelings of shame and fear; ED-related beliefs; and a lack of accessibility or availability of treatment [8]. The difficult accessibility of treatments is especially prevalent for people living in remote or rural areas [9]. The problem of insufficient mental health specialists able to provide therapies for Eds has also been recognized in Switzerland [10].

Disorder specific psychotherapy (cognitive behavioral therapy [CBT] and interpersonal psychotherapy [IPT]) have proven to be successful in reducing binge-eating symptomatology in the short and long term (e.g. [11–15]). Several studies have shown that among different BED treatment approaches (e.g. CBT and IPT) and settings (group or individual), up to 79% of the patients benefit from the therapy and show abstinence from binge-eating at the end of the active treatment [11, 16]. Maintenance of the therapy success in follow-up periods from 12 months up to five years has been proven in different studies from our group [13, 15]. The most thoroughly validated moderator for treatment success in BED is the reduction of binge-eating episodes by 65–70% within the first four treatment sessions (“rapid response”) (e.g. [13, 16]). A negative predictor of treatment success is over-evaluation of shape and weight. Also, the initial level of psychopathology seems to negatively influence treatment effects and it has been shown that therapeutic interventions are generally less successful with increasing duration of the ED [11, 16, 17]. The combination of pharmacotherapy and psychotherapy is not superior to psychotherapy alone and psychopharmacological medication in BED is still at an off-label-use level. Consequently, the spread and improvement of psychological treatments for BED is necessary [1] and Internet-based guided self-help (GSH) treatments are valuable treatment alternatives in BED therapy [1, 11, 18, 19].

The recently published NICE guidelines for the recognition and treatment of EDs strongly recommend GSH as the initial treatment provided in a stepped care approach [1]. CBT-based GSH treatments – in comparison to other BED related GSH – show the most pronounced positive outcomes [11, 18]. CBT GSH has been showed to be more effective compared to a waitlist control group, an unspecific therapy, and a weight-loss program. Abstinence rates of binge-eating in disorder-specific GSH were up to 64% with a significant reduction of psychopathology after treatment and at 12-month follow-up [20].

Modern technology, such as the Internet, has opened interesting possibilities for treatment delivery. These new approaches are advantageous since they are time- and location-independent, can be accessed anonymously, and thus might reduce feelings of shame and fear. They also require less implementation efforts and could be more cost-effective (less resources and infrastructure needed) than face-to-face interventions [21]. Several authors such as Aardoom et al. [22], Dölemeyer et al. [23], and Schlegl et al. [24] summarized the potential of technology-based interventions in comprehensive systematic reviews. Aardoom et al. [22] identified 21 studies on Internet-based treatments for EDs. Overall, they ascribed a high efficacy to these programs. Internet-based interventions appeared to be superior to waitlist conditions, e.g. in reducing ED pathology and binge-eating frequency, especially in individuals with less co-morbid disorders and in those suffering from binge-eating in contrast to restrictive ED symptomatology. Moreover, patients with BED showed better outcomes than patients with BN. In addition, the guidance of a therapist (e.g. via email) seems to increase the positive effects of Internet-based treatments [22]. Most of the investigated CBT-based GSH Internet-based programs are in English. Currently, there are only few studies on programs in German or French language with German- or French-speaking BED patients. The French-speaking program “Salut BED” has shown high acceptance and promising results in threshold and subthreshold BED patient as well as in obese BED patients. After six months of active Internet-based intervention, a significant reduction of binge-eating episodes, improved body image, higher psychological health, and better quality of life was observable after treatment and at six-month follow-up compared to a waitlist control group [25, 26]. “INTERBED” [27] is a German-speaking CBT-based program that has recently been evaluated in a multicenter randomized trial [28]. In this study, face-to-face CBT has proven higher efficacy than Internet-based GSH in reducing binge-eating episodes and ED pathology at the end of four months of treatment and at six-month follow-up. While the face-to-face treatment led to faster and more pronounced effects, the Internet-based GSH program still proved to be effective. At the 1.5-year follow-up, group differences no longer existed. Regarding quality of life, BMI, and general psychopathology, no difference between groups was found at any measurement point [28].

Alongside the advantages of Internet-based programs, there are also caveats such as dropout rates with high variances of 5–77%, whereas compliance was enhanced by pretreatment face-to-face assessments and therapists guidance through emails. These preliminary findings are of importance, as compliance was associated with better outcomes regarding the reduction of ED symptomatology [11, 22, 29, 30]. In a review, Schlegl et al. [24] found evidence for the efficacy of guided computer and Internet-based interventions, somewhat less for AN and more for patients with BN. They emphasized that further research is needed to understand the optimal level of therapist guidance in terms of frequency and quality. The question of the professional level of the guides and the efficacy of such programs also needs to be evaluated in comparison with face-to-face or blended treatments. Furthermore, research should address predictors and mediators of treatment outcomes.

The present randomized controlled trial (RCT) adds to existing data on treatment efficacy of Internet-based treatments for BED as the duration of treatment is shorter and additionally the effect of two waitlist conditions is evaluated. The first group is a pure waitlist control group (CG 1), where patients receive short weekly messages with a link to a short questionnaire to assess the number of weekly binges but no additional information during the four weeks of waiting time. The second group is a placebo-inspired control group (CG 2) that is provided with weekly messages with the aim to induce positive expectations regarding the program and to establish a therapeutic relationship with the therapist. This group is based on previous evidence from psychotherapy research referring to the effect of positive expectation on treatment adherence, therapeutic relationship, and dropout rate and treatment success [31–36]. Inspired by research on depressive disorder [37], this study will attempt to actively induce placebo in terms of positive expectation effects. To our knowledge, this is the first time this technique will be used in BED research. It will allow us to investigate the influence of positive expectations on treatment outcome in BED in more detail. The placebo inspired CG will allow rigorous examination of the presumably efficient disorder specific treatment that is applied in this study, since the specific active treatment is comparable to the unspecific placebo intervention.

Moreover, we will be able to indirectly compare the results of this Internet-based treatment to past studies of our group based on a GSH program with a book [38] and a face-to-face treatment [12, 14]. These are all based on the same CBT manual in order to develop hypotheses about moderators and mediators of treatment outcome for future studies.

## Methods

### Aim and research question

This project aims at developing an Internet-based treatment for BED based on evidence-based CBT [39].

The primary goal is to test the feasibility and suitability of the program and to evaluate the treatment outcome (short-term and long-term) in comparison to a pure and a placebo-inspired waitlist control group (CG) (i.e. reduction of binge-eating episodes and ED pathology as primary outcome variables). We expect that the immediate treatment group (TG) shows a significantly larger improvement in treatment outcomes during the first four weeks of treatment compared to the combined pure and placebo-inspired CGs during the first four weeks of the waiting period. We further expect that all three groups exhibit comparable temporal courses of treatment outcomes in the short (during eight weeks of active treatment) and long term (during the six months of follow-up). Thus, we expect that the placebo-inspired CG shows no advantage in terms of treatment outcome compared to the pure CG and the TG during the treatment and/or the follow-up phase. Our secondary goal deals with moderators and mediators of treatment outcome such as commitment, emotion and impulse regulation capacities, body image or interpersonal problems, depressive symptoms, and level of functioning.

The third goal of this study is to indirectly compare the present Internet-based treatment data with available data from: (1) a face-to-face therapy (group and single setting) in which the same therapeutic approach has been applied [12, 14]; and (2) from a conventional GSH program with a book relying on the same therapeutic concept [38].

The fourth goal is to explore the usability and acceptance of the program in order to gain information about users’ needs and specificities to improve interfaces of Internet-based treatments for vulnerable user groups such as patients with a mental disorder. We evaluate treatment integrity of therapists and treatment adherence of patients in order to increase the internal validity of our findings. The contents of the written feedback of the therapists are evaluated and we rate their fidelity with treatment guidelines. Patients’ compliance with the treatment is assessed using measures such as the time they spent with the program and by the number of exercises which are edited according to treatment suggestions.

### Development of the program

When developing an Internet-based GSH program, it is vital to provide a system with a good usability that supports commitment and motivation. This means users should be able to operate the GSH program as easily and intuitively as possible so they can focus on the content of the GSH program and do not need to spend time and effort to figure out how to operate the system. Moreover, it is important that users understand the content and instructions of the GSH program by themselves, with minimal assistance from a therapist. These circumstances impose several requirements in the form and content of a treatment as well as for the interaction design of the user interface of the GSH program. Since Internet-based treatments of mental disorders are quite novel, only little research exists on how usability affects users of such a program (e.g. outcome of a treatment, dropout rate, satisfaction with the treatment). The study by Currie et al. [40] is one of the few examples that took usability into account during the development of an Internet-based CBT program designed to reduce symptoms of emotional distress in students. Three cycles of usability testing including feedback from participants as well as from the counselling center staff lead to both structural (e.g. shortening sections) and stylistic (e.g. aesthetic features) changes, which resulted in a user-friendly platform [40].

To provide the best possible user experience, we aimed to develop a user-friendly Internet-based GSH program that is tailored to the needs of its end-user (in our case BED patients). To do so, the development of our program was driven by a user-centered design (UCD) approach. This means that during the entire development process we laid the focus on the needs, requirements, and limitations of our users. This was done through interviews, low-fidelity prototypes, observation of task-based walk-throughs, and think-aloud usability testing performed by users on functional prototypes. This allowed us to adapt the interface to our users, thereby ensuring a high usability [41].

We decided to apply Garrett’s [42] UCD model (elements of user experience) as a framework to guide the development process and to organize our project phases [43]. Following this procedure led to a fully developed and functional prototype/concept, which was subsequently programmed, evaluated and used to treat BED patients. We conducted extensive end-users research by reviewing literature on the clinical picture of BED, interviewing psychotherapists specialized on the treatment of BED, examining video footage of BED therapy sessions, and surveying *N* = 53 participants with a clinically relevant BED diagnosis (based on Eating Disorder Examination, EDE-Q scores) [44]. The information was used to develop three different personas (i.e. user archetypes used to help guide decisions about the design and features of the GHS program) [45] and to generate usage scenarios to specify the basic functionalities and content requirements of our tool.

### Sample and recruitment

**Fig. 1:**
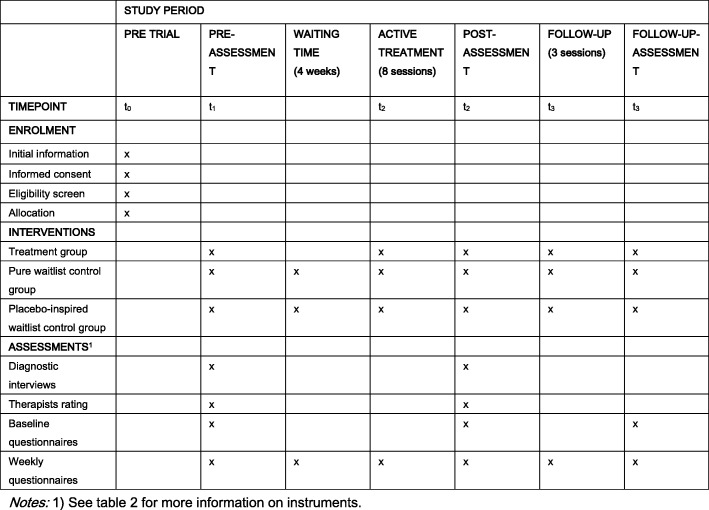
Interventional trials (SPIRIT) figure - schedule of enrolment, interventions, and assessments

Sixty participants with the primary diagnosis of a BED, that satisfy our inclusion criteria and aged 18–70 years, are randomly allocated (permuted block design; [46]) to three groups: 20 participants will be assigned to the treatment group (TG); 20 participants to a pure waitlist control group (CG 1); and 20 to the placebo-inspired waitlist control group (CG 2). No blinding procedure is applied; allocation is done by one of the primary authors (AW). All patients are required to give informed consent about the study’s procedure (e.g. diagnostic interview and questionnaires pre, post, and follow-up) and commit to follow the contents and guidance during the Internet-based sessions. The only exclusion criteria are pregnancy, the presence of another serious psychological or medical condition that warrants priority treatment, current drug or alcohol abuse, and the lack of sufficient German language or technical skills to work with the program (both self-reported). Recruitment of participants will be promoted via public advertisements and media as well as via cooperating clinical experts. The schedule (SPIRIT) of the trial is presented Fig. 1.

### Procedure

Altogether the active treatment phase including pre and post-diagnostic lasts approximately 11 weeks (plus four weeks in the control groups). The three follow-up sessions take place one, three, and six months after the last session of the active treatment. All exercises are an integral part of the sessions and mandatory. The patients are invited to work on the different exercises on an individual basis also between sessions.

Each patient is accompanied and guided by one of seven therapists that received a specific training in delivery of the current Internet-based treatment. All therapists are postgraduate psychologists or psychotherapists at the Center for Psychotherapy at the University of Fribourg and are continuously supervised by the study leaders (SM, AW). The therapists provide a weekly written feedback on the exercises via a communication system that is built into the GSH program. Patients can proceed to the next session after completing all parts of the current session along with the related exercises. The next session will then be unlocked for the patient. After finishing a session, patients will have to wait 7–10 days before they can proceed with the next session.

Since dropouts are a major problem in Internet-based treatments for EDs (up to 77%), our GSH program features several measures to support the compliance: a continuous personal contact to a therapist (face-to-face or telephone diagnostic assessment at the beginning of the program and messaging through the build-in communication system during the following three days after each session’s completion); a clear structure of the program (which has been shown to be beneficial in BED self-help programs) (e.g. [23]); and a conjoint scheduling. In addition, the introductory session provides patients with all necessary information to work smoothly through the program. Finally, the first two sessions include several interventions to develop and maintain sustained motivation. Altogether, this procedure aims at supporting the working alliance which has been positively related with the outcome in Internet-based treatments for BED [47].

The therapists that accompany the patients during the GSH program will be trained to provide feedback according to standardized topics and text passages, based on a previous book and email-based BED treatment approach of the outpatient clinic of the research group [38]. These topics are then customized to the patient’s individual needs. The communication between therapists and patients via the built-in communication system will be continuously supervised by the first two authors (SM, AW). Treatment integrity of randomly selected communication (i.e. written contact between therapist and patients) will be evaluated by independent raters according to the previously applied procedures in face-to-face treatments of the group [14, 15].

### Control groups (CG)

After having given informed consent, patients are randomly assigned to the TG (immediate start with the treatment) or either of the CGs. The waiting time in CG 1 (pure waitlist group) and CG 2 (placebo-inspired waitlist group) lasts four weeks. CG 2 will receive standardized weekly messages during their four-week waiting period. These messages include induction of positive expectations regarding program participation, a summary of the current evidence of BED treatment and benefits of such a standardized, evaluated treatment, quotes from former patients, and motivating words. This information does not contain any form of deception. The pure waitlist group will wait exclusively for four weeks. Both CGs fill in a short questionnaire to assess core BED symptomatology (i.e. binge-eating episodes) and to monitor changes in mood. After the waiting period, patients of both CGs start with the Internet-based GSH program.

### Material and measures

The content of the eight sessions and three follow-up sessions is all derived from established and evaluated treatment material [12–15, 39, 48, 49].

In Table 1, an overview of the main contents of each session is given.

**Table 1:** Main content of therapy sessions

Session	Content
0	Introduction to the program
1	Individual etiological model of BED
	Self-observation of eating behavior
2	Motivation
	Goal attainment scale
3	Regular eating
	Analyzing binge-eating episodes with the ABC-model
4	Developing strategies to overcome binge-eating (trigger and reaction control)
5	Positive/enjoyable activities
	Working with emergency cards
6	Expanding strategies
	Dysfunctional thoughts
7	Dysfunctional thoughts and body image
	Reduction of overweight
8	Setting longer-term goals
	Coping with future difficulties
	Relapse prevention
Follow-up sessions 1–3	Further goals
	Coping with current difficulties
	Relapse prevention

Table 2 gives an overview of all instruments that will be applied during the study. Primary outcome variables are number of binge-eating episodes and ED pathology. Secondary outcome variables are reduction of depressive symptoms and increase of the level of functioning. Moreover, all exercises that the patients edited during the sessions as well as all communication between the therapist and the patients will be stored and evaluated (e.g. regarding treatment integrity).

**Table 2:** Instruments

Instrument	Description/Construct	Time point
Interviews		
Diagnostic interview for psychiatric disorders, short version (Mini-DIPS; [53])	Structured interview to assess psychiatric disorders according DSM-5. Duration: approx. 45 min.	t_1_, t_2_
Eating Disorder Examination (EDE; German version [54])	Structured interview to assess eating disorder pathology Duration: approx. 45 min.	t_1_
Therapists ratings		
Clinical global impression scale [55]	Measure of symptom severity, treatment response and the efficacy of treatments in patients with mental disorders. 7 items rated by the clinician.	t_1_, t_2_
Self-report questionnaires		
Sociodemographic questions (own items)	Sociodemographic questions (gender, age, nationality, sick leave days, prior treatments etc.). 35 items.	t_1_
Patient’s expectancies regarding the therapy (own items)	Questions to assess attitude and expectations of the patients towards the internet-based treatment. 5 items.	t_1_
Work and Social Adjustment Scale (WSAS; [56])	Questionnaire to assess functional impairment (in the area of work, family and social functioning). 5 items.	t_1_, t_2,_ t_3_
Rosenberg Self-Esteem Scale (RSES; [57]	Measurement of self-esteem. 10 items.	t_1_, t_2,_ t_3_
Ein Messinstrument zur Erfassung subjektiver Kompetenzerwartungen (ASKU; [58])	Assessment of subjective expectation of competency. 3 Items.	t_1_, t_2,_ t_3_
Sensitivity to Rejection Scale (MSR; [59])	Scale to measure sensitivity to rejection, submissiveness and ability to deal with threat and hostility. 9 items.	t_1_
Beck Depression Inventory II (BDI-II; German version [60])	Measure of the severity of depressive symptoms. 21 items.	t_1_, t_2,_ t_3_
Beck Depression Inventory Fast Screening (BDI-FS; [61])	Short assessment of depressive symptoms (excluding somatic symptoms). 7 items.	Weekly during waiting time and treatment
Beck Anxiety Inventory (BAI; German version [62])	Measures of the severity of anxiety symptoms. 21 items.	t_1_, t_2,_ t_3_
Eating Disorder Examination-Questionnaire (EDE-Q; German version [63])	Assessment of eating disorders pathology during the past 28 days. 4 scales: eating concerns, weight concerns, restraint eating, shape concerns. 28 items.	t_1_, t_2,_ t_3_
Dutch Eating Behavior Questionnaire (DEBQ; German version [64])	Questions to assess emotional eating. 10 items.	t_1_, t_2,_ t_3_
Weekly Binges Questionnaire (WBQ; [12])	Questions to assess frequency of binge-eating episodes and regularity of eating behavior. 7 items.	Weekly during waiting time and treatment
Thought-Shape Fusion Questionnaire (TSF; [65])	Assessment of body-related cognitive distortions triggered by fattening/forbidden foods. 17 items.	t_1_, t_2,_ t_3_
Food Craving Questionnaire (FCQ trait, short version; [66])	Measure of the intensity of food cravings on a multidimensional level. 15 items.	t_1_, t_2,_ t_3_
Difficulties in Emotion Regulation Scale (DERS; German version [67])	Assessment of central aspects of affective experiences and emotion processing. 36 items.	t_1_, t_2,_ t_3_
Negative Effects Questionnaire (NEQ; [68])	Questionnaire to assess potentially adverse and unwanted events in psychological treatments. 32 items.	t_2,_ t_3_
Adapted version of the Working Alliance Inventory (WAI-SR; [69])	Assessment of main aspects of therapeutic alliance, resource activation, problem actualization, session outcome. 23 items.	Weekly during treatment
Final evaluation of the internet-based treatment (own items)	Assessment of self-reported estimation of therapy outcome. 9 items.	t_2,_ t_3_
Usability		
System Usability Scale (SUS; [70])	10 items to assess usability and user friendliness.	t_2_
Appreciation [71]	Three-item scale to assess to what extent the media experience was considered meaningful, thought-provoking, and moving.	Weekly during treatment
Emotional Engagement with Character [72]	Four-item subscale to assess emotional engagement with a character; used to measure the engagement with the character of a fictional former patient, whose presence guided the patient through the Internet-based treatment sessions.	t_2_

### Statistical analysis

In model 1 we test whether treatment outcomes improve during the active treatment phase across (i.e. pooled for) all three groups to test for the overall efficacy of the GSH program. This model thus contains one within-subjects factor (pre-post). In model 2 we test whether the temporal course of GSH program’s outcomes during active treatment and during follow-up varied among the three groups (TG, CG1, and CG2). We will apply a two-way mixed analysis of variance with group (three levels) as between-subjects and time (pre-post or post-follow-up) as within-subjects factors, whereby we are interested in the interaction between group and time.

Power analyses: based on prior research data (e.g. [25]), we expect an 18% dropout rate for our power estimations. The correlation (rho) of the number of weekly binges between the two time points is estimated to be around 0.6 (using data published in [14]). Here we assume a slightly more conservative value of rho = 0.5. Based on alpha = 0.05 (two-sided), beta = 0.2, a medium effect size (Cohen’s f = 0.25), and a dropout rate of 18%, the estimated sample size is 34 based on model 1 and 42 (14 per group) based on model 2. Taking the larger of these two values (42) this value was subsequently increased to 60 (20 per group) to be on the safe side with respect to the required sample size.

Analyses based on the secondary goal will include moderators and mediator models.

Analyses for the third (compare the present Internet-based treatment with available data from a face-to-face therapy and conventional GSH program with a book) and fourth goal (explore usability) are of exploratory nature and will thus use descriptive statistics such as means, standard deviations, and percentages.

Multilevel models will be used to analyze the data of the first and second goals [50], including multilevel structural equation models (for mediator models in second goal [51]).

### Ethical considerations

Ethics approval was given by the Ethics Committee of the canton Bern (Switzerland). Project-ID: 2017–00102. All participants will be informed in accordance with the study protocol approved by the Ethics Committee (clinical study protocol version 4, 06.07.2017).

## Discussion

The present RCT evaluates a newly developed CBT-based GSH program delivered via the Internet. Next to accessibility and independence of time and location, another benefit of such a manualized program is the clear focus on the disorder-specific factors in treatment. The results of the present study will allow recommendations regarding suitabilty and efficacy of low-threshold interventions for patients suffering from BED [17].

We postulate several advantages for this clinical trial. First, the treatment duration of approximately 11 weeks (including diagnostics) is shorter than in the studies by Carrard et al. [25] (6 months) and de Zwaan et al. [27] (4 months). In our face-to-face treatment studies, a longer treatment period has been associated with a higher dropout rate of 30% [14] compared to 13% dropouts in a shorter, eight-week treatment [12]. Carrard et al. [26] reported dropout rates of 9% (2/22 participants in the Internet-based treatment group) and de Zwaan et al. [28] of 19.1% (17/89 participants in the GSH intervention). Second, we will apply an Internet-based version of our original BED treatment program [39], which has been systematically evaluated in RCTs in face-to-face group settings. This will enable us to indirectly compare the efficacy of the Internet-based BED treatment with data from previous studies [12–15]. Third, and for the first time in BED research, the present study investigates the role of interventions to increase positive expectations (placebo) regarding the upcoming treatment.

The efficacy, usability, and adoption of the developed program will be evaluated through interviews, questionnaires, and analysis of interaction logs. In respect of psychotherapy research, the present study provides the possibility of examining the efficacy of a standardized disorder-specific treatment programm in comparison to a pure waiting period and a placebo-inspired intervention. Even if we make the rather conservative assumption that the specific intervention during active treatment is the “verum” that results in positive outcome and the “placebo” is not effective, we could nevertheless assume that the placebo-inspired CG shows advantages in terms of a faster positive development during treatment due to higher positive expectations and a more established therapeutic relationship already at the beginning of the active treatment. Such a result would refer to an “add-on effect” of a placebo-inspired waiting period or in other words of a preparation phase before a disorder-specific treatment, which would be relevant for clinical practice. The comparison of the present GSH program with established face-to-face therapy and a book-based GSH program, as well as the investigation of moderating and mediation variables, will improve the understanding of differential indications in BED treatment.

This research will also help to understand the specific interaction traits of users following an Internet-based treatment and to gather requirements for user interfaces that support their needs and reduce the risk of therapy drop-out. Another expected contribution of this research in the field of human–computer interaction is the exploration of mechanisms to adapt automatically the content or alerts of the therapy according to the patient specificities, towards user-induced adaptation [52] and personalized therapy.

To sum up, the development of an Internet-based BED treatment increases the feasibility to recruit larger patient groups with less subject burden in terms of time and loss of anonymity for patients. Better knowledge about the effect and the determinants of the effect of Internet-based treatments might potentially lead to more cost-effective approaches to intervention. From a scientific point of view, larger samples from the general population suffering from BED will allow more fine-grained research on moderators and mediators of change in the future. This information will enrich etiological models and might stimulate the development of additional treatment modules, which are easily implemented in the Internet-based treatment (e.g. training of emotion regulation, emotion regulation in interpersonal relationships, etc.).

The Internet-based GSH program could be adapted for other EDs such as AN and BN or individuals suffering from EDs not further specified. It could also prove useful for groups of patients with specific profiles such as non-responders, adolescents, and older patients, or as a support during transfer from inpatient to outpatient treatment (especially when the patient has to wait for a specialized BED outpatient treatment). The question of the intensity and quality of the psychotherapeutic support/guidance (e.g. via email contact) will also be addressed and whether guidance could also be provided by non-specialists (i.e. a person without a psychotherapeutic background). After investigating efficacy under controlled research conditions, the GSH program will be implemented into daily clinical practice and its effectiveness evaluated.

## Trial status

The study protocol has been approved by the Ethics Commission on 27 July 2017. Trial registration was completed on 14 September 2017. Recruitment started on 30 October 2017 and will presumptively end on 30 June 2018.

Protocol version number: 4.

Protocol version date: 06.07.2017.

## Abbreviations

BED: Binge-eating disorder
CBT: Cognitive behavioral therapy
CG: Control group
ED: Eating disorder
GSH: Guided self-help
IPT: Interpersonal psychotherapy
TG: Treatment group
